# New plastome structural rearrangements discovered in core Tillandsioideae (Bromeliaceae) support recently adopted taxonomy

**DOI:** 10.3389/fpls.2022.924922

**Published:** 2022-08-01

**Authors:** Sandra I. Vera-Paz, Daniel D. Díaz Contreras Díaz, Matthias Jost, Stefan Wanke, Andrés J. Rossado, Rebeca Hernández-Gutiérrez, Gerardo A. Salazar, Susana Magallón, Eric J. Gouda, Ivón M. Ramírez-Morillo, Sabina Donadío, Carolina Granados Mendoza

**Affiliations:** ^1^Departamento de Botánica, Instituto de Biología, Universidad Nacional Autónoma de México, Mexico City, Mexico; ^2^Posgrado en Ciencias Biológicas, Universidad Nacional Autónoma de México, Mexico City, Mexico; ^3^Institut für Botanik, Technische Universität Dresden, Dresden, Germany; ^4^Laboratorio de Sistemática de Plantas Vasculares, Facultad de Ciencias, Universidad de la República, Montevideo, Uruguay; ^5^Department of Evolution, Ecology, and Organismal Biology, University of California, Riverside, Riverside, CA, United States; ^6^Botanical Garden, Utrecht University, Utrecht, Netherlands; ^7^Herbario CICY, Centro de Investigación Científica de Yucatán, A.C. (CICY), Yucatán, Mexico; ^8^Instituto de Botánica Darwinion (CONICET-ANCEFN), Buenos Aires, Argentina

**Keywords:** ancestral state reconstruction, evolutionary rate shifts, gene translocation, inverted repeats (IRs), inversions, plastome, phylogenetic informativeness

## Abstract

Full plastome sequences for land plants have become readily accessible thanks to the development of Next Generation Sequencing (NGS) techniques and powerful bioinformatic tools. Despite this vast amount of genomic data, some lineages remain understudied. Full plastome sequences from the highly diverse (>1,500 spp.) subfamily Tillandsioideae (Bromeliaceae, Poales) have been published for only three (i.e., *Guzmania*, *Tillandsia*, and *Vriesea*) out of 22 currently recognized genera. Here, we focus on core Tillandsioideae, a clade within subfamily Tillandsioideae, and explore the contribution of individual plastid markers and data categories to inform deep divergences of a plastome phylogeny. We generated 37 high quality plastome assemblies and performed a comparative analysis in terms of plastome structure, size, gene content and order, GC content, as well as number and type of repeat motifs. Using the obtained phylogenetic context, we reconstructed the evolution of these plastome attributes and assessed if significant shifts on the evolutionary traits’ rates have occurred in the evolution of the core Tillandsioideae. Our results agree with previously published phylogenetic hypotheses based on plastid data, providing stronger statistical support for some recalcitrant nodes. However, phylogenetic discordance with previously published nuclear marker-based hypotheses was found. Several plastid markers that have been consistently used to address phylogenetic relationships within Tillandsioideae were highly informative for the retrieved plastome phylogeny and further loci are here identified as promising additional markers for future studies. New lineage-specific plastome rearrangements were found to support recently adopted taxonomic groups, including large inversions, as well as expansions and contractions of the inverted repeats. Evolutionary trait rate shifts associated with changes in size and GC content of the plastome regions were found across the phylogeny of core Tillandsioideae.

## Introduction

Complete plastome sequences for more than 10,300 vascular plant species have been made publicly available at the National Center for Biotechnology Information until March 2022 (NCBI, only verified and circular DNA sequences considered). This wealth of genomic resources has not only greatly improved our knowledge of the plant tree of life and the evolutionary processes that shape plant diversity (e.g., Ruhfel et al., 2014; Li et al., 2021), but also eased the study of patterns of plastome diversity and evolution across plant lineages (e.g., Jansen et al., 2007; Zhu et al., 2016).

Despite the vast number of currently available vascular plant plastomes, most of which belong to flowering plants (Mower and Vickrey, 2018), plastome diversity for certain angiosperm families remains poorly explored. Full plastome sequences from the highly diverse monocot family Bromeliaceae (Poales, 3,714 spp.; Gouda et al., 2018) have been published for only 13 species and one hybrid cultivar (Nashima et al., 2015; Redwan et al., 2015; Poczai and Hyvönen, 2017; Paule et al., 2020; Chávez-Galarza et al., 2021; Möbus et al., 2021; Wu et al., 2022), representing three (Bromelioideae, Puyoideae, and Tillandsioideae) out of the eight Bromeliaceae subfamilies (Givnish et al., 2007). In 2015, two independent research groups published the first Bromeliaceae plastomes, both from pineapple (*Ananas comosus*, Bromelioideae; Nashima et al., 2015; Redwan et al., 2015), and one year later a third *A. comosus* plastome was published by Liu et al. (2016). Poczai and Hyvönen (2017) published the Spanish moss (*Tillandsia usneoides*, Tillandsioideae) plastome, reporting a highly conserved structure, gene content and gene order relative to the plastome of the distantly related *A. comosus*. Additional plastomes were published for three Bromelioideae species of the *Fascicularia-Ochagavia* group (i.e., *Fascicularia bicolor*, *Ochagavia carnea*, and *Ochagavia elegans*) and *Puya mirabilis* (Puyoideae) by Paule et al. (2020). These authors also reported overall conserved structural properties relative to the previously published *A. comosus* plastomes. However, two expansions of the inverted repeat (IR) at the border of the IR and the large single copy (LSC) were identified for *F. bicolor* and *O. carnea*, where the *rps*3 gene of *F. bicolor*, and the *rps*3, *rpl*14, *rpl*16, and partial *rps*8 genes of *O. carnea* are translocated from the LSC to the IR relative to other Bromelioideae. Chávez-Galarza et al. (2021) and Möbus et al. (2021) published plastomes for *Tillandsia landbeckii* and for *Tillandsia espinosae*, *Tillandsia malzinei*, and *Tillandsia purpurea*, respectively, reporting similar structural and gene content properties to the previously published *T. usneoides* plastome. A recent phylogenetic study by Wu et al. (2022) focusing on the order Poales, contributed four additional Bromeliaceae plastomes, belonging to the subfamilies Bromelioideae (*Cryptanthus acaulis* and *Neoregelia* sp.) and Tillandsioideae (*Guzmania conifera* and *Vriesea* × *poelmanii*), but no further comparison to other previously published Bromeliaceae plastomes was provided.

The present study focuses on core Tillandsioideae, a clade within subfamily Tillandsioideae from which over 1,500 spp. have been described (Gouda et al., 2018). Classification of subfamily Tillandsioideae was significantly restructured in the taxonomic revision of Barfuss et al. (2016), where monophyletic units, identified on a molecular phylogeny, were circumscribed by synapomorphic combinations of diagnostic morphological characters, including sepal symmetry; corolla morphology; petal connation/conglutination; presence/absence of petal appendages; filaments connation and adnation to petals; pollen type; ovary position; stigma type; presence/absence of ovule appendages and, when present, their size relative to the ovary; and seed appendage type. The 22 currently recognized genera (including *Waltillia*; Leme et al., 2017) are classified into four tribes: Catopsideae (18 spp. and 1 genus), Glomeropitcairnieae (2 spp. and 1 genus), Tillandsieae (1,094 spp. and 8 genera), and Vrieseeae, the latter further divided in subtribes Vrieseinae (299 spp. and 4 genera) and Cipuropsidinae (117 spp. and 8 genera; Barfuss et al., 2016; species numbers according to Gouda et al., 2018). As recovered by Barfuss et al. (2016), tribes Catopsideae (*Catopsis*) and Glomeropitcairnieae (*Glomeropitcairnia*) conform the non-core Tillandsioideae clade, whereas the core Tillandsioideae clade is integrated by tribes Tillandsieae and Vrieseeae, and includes the genera *Alcantarea* (46 spp.), *Barfussia* (5 spp.), *Cipuropsis* (3 spp.), *Goudaea* (2 spp.), *Gregbrownia* (4 spp.), *Guzmania* (217 spp.), *Jagrantia* (1 sp.), *Josemania* (5 spp.), *Lemeltonia* (7 spp.), *Lutheria* (4 spp.), *Mezobromelia* (6 spp.), *Pseudalcantarea* (3 spp.), *Racinaea* (83 spp.), *Stigmatodon* (20 spp.), *Tillandsia* (770 spp.), *Vriesea* (231 spp.), *Wallisia* (5 spp.), *Waltillia* (2 spp.), *Werauhia* (96 spp.), and *Zizkaea* (1 sp.). Additional species and genera complexes were recognized by Barfuss et al. (2016), but formal taxonomic changes for these groups await for key taxa to be sampled (e.g., the *Cipuropsis-Mezobromelia* complex) or increased phylogenetic support to be achieved (e.g., *Tillandsia biflora* complex).

Tillandsioideae phylogenetics, and in general in Bromeliaceae, has heavily relied on Sanger sequenced data, either from plastid, nuclear-ribosomal, or nuclear low copy loci that have been employed from deep to shallow taxonomic levels (Terry et al., 1997; Barfuss et al., 2005, 2016; Granados Mendoza, 2008; Chew et al., 2010; Versieux et al., 2012; Donadío, 2013; Castello et al., 2016; Pinzón et al., 2016; Leme et al., 2017; Kessous et al., 2020; Donadío et al., in press). Recent studies have applied with great success Next Generation Sequencing (NGS) strategies to Tillandsioideae phylogenetics. Machado et al. (2020) used genome skimming to mine plastid protein-coding genes and the low copy nuclear *phy*C gene for a comprehensive sample of tribe Vrieseeae and a representation of tribe Tillandsieae. Then, Loiseau et al. (2021) expanded the plastid genomic dataset of Machado et al. (2020), by mining additional nuclear and mitochondrial loci for an increased taxon sampling. Lastly, Yardeni et al. (2022) explored the utility of two nuclear target capture kits to resolve phylogenetic relationships in Bromeliaceae, including 56 species of the subfamily Tillandsioideae.

Bromeliaceae are known for their low substitution rates compared to other Poales (Smith and Donoghue, 2008). Therefore, phylogenetic resolution and support in core Tillandsioideae could benefit from the inclusion of NGS based data, as demonstrated by the studies of Machado et al. (2020); Loiseau et al. (2021), and Yardeni et al. (2022). To our knowledge, no study has explored plastome diversity and evolution on a representative taxon sampling of the core Tillandsioideae, since full plastome sequences are only available for three (i.e., *Guzmania*, *Tillandsia*, and *Vriesea*) of the 22 currently recognized genera of the entire subfamily.

In the present study, we generate high quality plastome assemblies and perform a comparative analysis in terms of plastome structure, size, gene content and order, GC content, as well as number and type of repeat motifs. Additionally, we explore the contribution of individual plastid markers for resolving deeper-level divergences in the obtained plastome-based phylogenetic framework of the core Tillandsioideae and assess the phylogenetic informativeness (PI) of plastid data categories, such as coding and non-coding loci. Based on the retrieved phylogenetic context, we reconstruct the evolution of selected plastome attributes and determine if significant shifts on their evolutionary rates have occurred during the evolution of the core Tillandsioideae.

## Materials and methods

### Taxon sampling

Plastomes of 35 species of Tillandsioideae were newly sequenced and assembled for this study. The sampling was expanded with genome skimming data publicly available at the NCBI Sequence Read Archive (SRA), resulting in two additional, complete, and newly assembled Tillandsioideae plastomes (Supplementary Material 1). Species were selected to best represent main lineages within core Tillandsioideae, including 14 out of 20 of its currently recognized genera. Tribe Tillandsieae was represented by species of the genera *Barfussia* (1 sp.), *Guzmania* (1 sp.), *Lemeltonia* (1 sp.), *Pseudalcantarea* (2 spp.), *Racinaea* (1 sp.), *Tillandsia* (21 spp.), and *Wallisia* (1 sp.). Sampling within genus *Tillandsia* included representatives of the subgenera *Aerobia* (1 sp.), *Anoplophytum* (1 sp.), *Diaphoranthema* (1 spp.), *Phytarrhiza* (1 sp.), *Pseudovriesea* (2 spp.), *Tillandsia* (7 spp.), and *Viridantha* (1 sp.), as well as the species complexes of *Tillandsia australis* (1 sp.), *T. biflora* (2 spp.), *Tillandsia disticha* (1 sp.), *Tillandsia gardneri* (1 sp.), *T. purpurea* (1 sp.), and *Tillandsia sphaerocephala* (1 sp.). Tribe Vrieseeae subtribe Cipuropsidinae was represented by one species for each of the genera *Goudaea*, *Lutheria*, *Mezobromelia*, *Werauhia*, and *Zizkaea*, whereas tribe Vrieseeae subtribe Vrieseinae was represented by the genera *Alcantarea* (1 sp.), and *Vriesea* s.s. (2 spp., *sensu* Machado et al., 2020). *Catopsis sessiliflora* of tribe Catopsideae (non-core Tillandsioideae) was used to root the phylogenetic tree, following phylogenetic relationships reported by Barfuss et al. (2016). The six non-sampled core Tillandsioideae genera include Andean *Gregbrownia*, *Josemania* from Costa Rica, Panama, Colombia, Ecuador and Peru, *Cipuropsis* from the northern Andes to Venezuela, Trinidad, and the Guianas, *Jagrantia* from Central America to Colombia, and Brazilian *Stigmatodon*, and *Waltillia* (Barfuss et al., 2016; Leme et al., 2017). Taxonomy follows Barfuss et al. (2016) and Gouda et al. (2018).

### DNA extraction

Genomic DNA was extracted from silica-gel dried leaf tissue using the DNeasy Plant Pro Kit (Qiagen) or a standard CTAB method following Doyle and Doyle (1987), the latter modified to include a treatment with RNase A (Qiagen, 100 mg/ml) and proteinase K (Thermo Scientific,™ 1 mg/ml). DNA concentration was quantified with a Qubit™ fluorometer v.3.0 (Invitrogen,™ Thermo Fisher Scientific), using the Qubit™ dsDNA BR Assay Kit (Invitrogen,™ Thermo Fisher Scientific). DNA purity was assessed with a Nanodrop 2000 spectrophotometer (Thermo Scientific™), ensuring 260/280 ratios ≥1. The molecular weight of the genomic DNA was evaluated in 1% agarose test gels, run for 50 min at 85 V and 500 mA.

### DNA library preparation, enrichment, and sequencing

Herein newly sequenced plastomes were generated from raw data derived from a Hyb-Seq project that uses the universal probe kit for angiosperms of Buddenhagen et al. (2016), modified to include additional sequences of Bromeliaceae representatives. Library preparation, enrichment and sequencing was performed by Daicel Arbor Biosciences^1^ as follows. Samples were quantified *via* a spectrofluorimetric assay with PicoGreen (ThermoFisher Scientific). Genomic DNA was sheared with a Qsonica Q800 ultrasonicator to produce insert lengths of approximately 300–800 nt. Library preparation followed a proprietary modification by Daicel Arbor Biosciences of the KAPA HyperPrep kit (Roche). Custom unique dual-index combinations were added to each sample *via* 6–10 cycles of PCR amplification. The indexed libraries were quantified with both a spectrofluorimetric assay and a quantitative PCR assay using a KAPA Library Quantification Kit (Roche). Capture pools were prepared from 200 ng of 12 or 13 libraries per reaction and each capture pool was reduced to 7 μL by vacuum centrifugation. Capture was performed following the myBaits v.5 protocol^2^ with an overnight hybridization and washes at 60°C. Post-capture, the reactions were amplified for 12 cycles and were quantified with spectrofluorimetric and quantitative PCR assays. The captures were pooled in approximately equimolar ratios based on the number of libraries in each capture. A second equimolar pool of non-captured libraries was prepared. The captured and non-captured pools were combined at a 70:30 ratio. Samples were sequenced on the Illumina NovaSeq 6000 platform on S4 PE150 lanes to approximately 2 Gbp per library.

### Plastome assembly and annotation

Two *de novo* assembly strategies were applied for a subset of samples (i.e., *Pseudalcantarea macropetala, Tillandsia imperialis*, *Tillandsia utriculata*, and *Vriesea sucrei*) in order to validate our methods. In the first strategy, paired reads of each accession were *de novo* assembled in CLC Genomics Workbench v.11.0,^3^ allowing for automatic word and bubble size, as well as an auto-detection of paired distances. Reads were mapped back to the contigs and the resulting read mapping files were visualized in Tablet v.1.21.02.08 (Milne et al., 2013) to evaluate correctness of the assembly, contig coverage and to detect potential mis-assemblies. Contigs of possible plastome origin were mined out of the full assembly *via* a custom BLAST in Geneious Prime^®^ 2021.2.2,^4^ with a max *E*-value = 1e−10 and a word size of 28, using a database (Supplementary Material 2) consisting of 47 complete angiosperm plastomes, including *A. comosus* (KR336549.1) and *T. usneoides* (KY293680.1), publicly available in GenBank. Further scaffolding was performed in Geneious Prime^®^.

The *de novo* assembler GetOrganelle v.1.7.5 (Jin et al., 2020) was used as our second assembly strategy, applying a K-mer gradient (-k 21,45,65,85,105) appropriate for 150 bp long paired data, up to 20 maximum extension runs, an automatically estimated word size and the default seed database (embplant_pt) to filter plastid-like reads. The generated plastomes were visualized in Bandage (Wick et al., 2015) and LSC, small single copy (SSC), and IR coverage was extracted from each corresponding graph segment. Of the two naturally existing isomeric plastome sequences (due to different orientations of the single copy regions; Palmer, 1983; Walker et al., 2015) we selected the one matching the published *A. comosus* (KR336549.1) plastid genome for downstream analyses. Once results from both assembly strategies were cross-validated, assemblies of the remaining samples were only performed with GetOrganelle as described above.

Annotations from *A. comosus* (KR336549.1) and *T. usneoides* (KY293680.1) were first automatically transferred to the target species *T. utriculata* (the type species of genus *Tillandsia*) and then manually inspected in Geneious Prime^®^. The annotated and curated plastome sequence of *T. utriculata* was subsequently used as reference to automatically transfer annotations to the remaining plastomes, which were also manually curated. The tRNAscan-SE web server^5^ (Lowe and Chan, 2016; Chan and Lowe, 2019) was used with default settings to confirm and, where necessary, adjust the predicted tRNA gene borders during automatic annotation. Newly sequenced and assembled plastomes can be found in GenBank under the accession numbers ON398129–ON398163, whereas newly assembled plastomes from NCBI SRA data can be found in the Third Party Annotation Section of GenBank under the accession numbers TPA: BK061352-BK061353.

### Plastomes comparison and repeat regions analyses

All assembled plastomes were compared for several attributes, including gene number, gene content, gene order, GC content, as well as overall, LSC, SSC, and IR size, and general structure in Geneious Prime^®^ 2022.0.2 using standard tools. Major inversions detected during manual curation of the alignment referred in the next section were confirmed with the LASTZ plugin v.1.02.00 of Geneious Prime^®^ using *C. sessiliflora* as target sequence and default settings. The MIcroSAtellite identification tool v.2.1 (MISA-web; Beier et al., 2017) was used for the detection of Simple Sequence Repeats (SSR), assuming a minimum number of repetitions of 10 for mononucleotide repeats, of six for dinucleotide repeats, and of five for tri-, tetra-, penta-, and hexanucleotide repeats, and 100 as maximum length of sequence between two SSRs to register as compound SSR. Tandem repeats were detected with Tandem Repeats Finder v.4.09.1 (Benson, 1999), applying default parameters. Both SSR and tandem repeat detection were performed on complete plastomes, distinguishing repeat motifs present in the IR regions, LSC and SSC.

### Tree reconstruction

Complete plastomes were aligned with MAFFT v.7.487 (Katoh and Standley, 2013) and subsequently trimmed to only include one of the two IR copies for each species in the final alignment. The alignment was partitioned by protein-coding genes, tRNAs genes, rRNAs genes, introns, and intergenic spacers (IGS). Inversions were identified by eyeballing and included: (1) a large inversion (28,343 bp) found in the two *Vriesea* species spanning genes *psb*D to *acc*D and a portion of each of the two flanking IGS; (2) a smaller inversion (2,222 bp) present in the two representatives of the *T. biflora* complex including the *acc*D gene and partial *rbc*L*-acc*D and *acc*D*-psa*I IGS; and (3) several minor inversions (<200 bp) distributed across the alignment. Poorly aligned and divergent regions were identified with Gblocks (Castresana, 2000), setting the allowed gap positions parameter to the option “all.” Inversions and poorly aligned regions were excluded from further analyses using the -E command of RAxML v.8.2.10 (Stamatakis, 2014), which generates modified matrix and partition files. Phylogenetic inference was performed on this modified partitioned matrix under Maximum Likelihood (ML) in the IQ-TREE web server^6^ with an edge-linked partition model, and an automatic selection of the best-fit substitution model for the partitions. Node support was estimated based on 1,000 replicates of the ultrafast bootstrap approximation (UFBoot).

### Estimation of phylogenetic informativeness

A PI (Townsend, 2007) analysis was carried out to assess the contribution of different plastid markers and data partitions in resolving the retrieved plastid phylogenetic relationships within core Tillandsioideae. The PI method (Townsend, 2007) quantifies the likelihood that a character changes at a certain point of a tree, remaining subsequently unchanged. This is estimated by comparing evolutionary changes across sites against an ideal change rate based on an ultrametric tree, where branches are proportional to evolutionary units. The tree resulting from the analysis detailed in the previous section was transformed to ultrametric with the function *chronos* of the R v.4.1.2 (R Core Team, 2021) package *ape* (Paradis and Schliep, 2019), setting the model of substitution rate variation among branches to be relaxed and the smoothing parameter lambda = 0. An arbitrary time scale from 0 at the tree tips to 100 at the root, and not from 0 to 1, was applied to avoid generating an ultrameric tree with branches of size close to zero that can cause problems in downstream analyses. Site substitution rates and net PI profiles of each locus were calculated in TAPIR^7^ using individual alignments corresponding to each data partition, along with the ultrametric tree. Maximum PI values and the time at which these values were reached were extracted to compare the phylogenetic utility of loci and data categories to inform the retrieved plastome phylogeny.

### Ancestral state reconstruction of plastome attributes

Ancestral state reconstruction was performed for 20 continuous plastome attributes in R (R Core Team, 2021). The studied attributes included size, GC content, as well as number of SSR and tandem repeats per one kb, considering both the entire plastome and each of its regions (i.e., LSC, SSC, and IR). For the repeat motifs, distinction was also made between coding and non-coding regions. Ancestral states and their 95% confidence intervals at the nodes of the ultrametric Tillandsioideae tree were estimated under a ML framework with the functions *fastAnc* of the package *phytools* v.1.0-1 (Revell, 2012). The attributes were mapped on the ultrametric tree with the function *contMap* of *phytools*.

Additionally, we evaluated the presence of evolutionary rate shifts through time and among lineages of the analyzed plastome attributes. For this, we transformed attribute values to logarithmic scale to normalize their distribution. We implemented a Bayesian Analysis of Macroevolutionary Mixtures (BAMM v.2.5.0; Rabosky, 2014), which estimates phenotypic evolutionary rate under a compound Poisson process throughout the phylogeny. Using reversible-jump Markov Chain Monte Carlo (rjMCMC), BAMM evaluates different models that vary in the number of shifts in the evolutionary rate of a phenotypic trait or attribute. As opposed to diversification rate shifts, modeling of phenotypic evolutionary rate shifts in BAMM^8^ does not require accounting for incomplete taxon sampling. In this case, BAMM considers trait data to estimate and evaluate the potential shifts in the rate of evolution of the target phenotypic trait across the provided phylogeny. Furthermore, our taxon sampling was designed to best represent the main lineages of the Core Tillandsioideae and, although additional plastome diversity might still to be uncovered, our study includes nearly all the existing full plastomes for the focal group. Therefore, our analyses include a dense representation of the known plastome diversity to date. Prior settings suitable for our data set were first explored with the R package BAMMtools (Rabosky, 2014). BAMM analyses were performed with the selected priors and run for 20 million generations. Chain convergence was examined with the package coda (Plummer et al., 2006) and an Effective Sample Size (ESS) of the MCMC ≥200 was ensured. BAMM output was analyzed with BAMMtools in R to extract the number and probability of shifts in the evolutionary rate for each plastome attribute. When a shift or set of shifts was detected, a Maximum *a posteriori* probability (MAP) configuration was generated to visualize the number and location of the shifts.

## Results

### Tillandsioideae plastomes attributes

**FIGURE 1:**
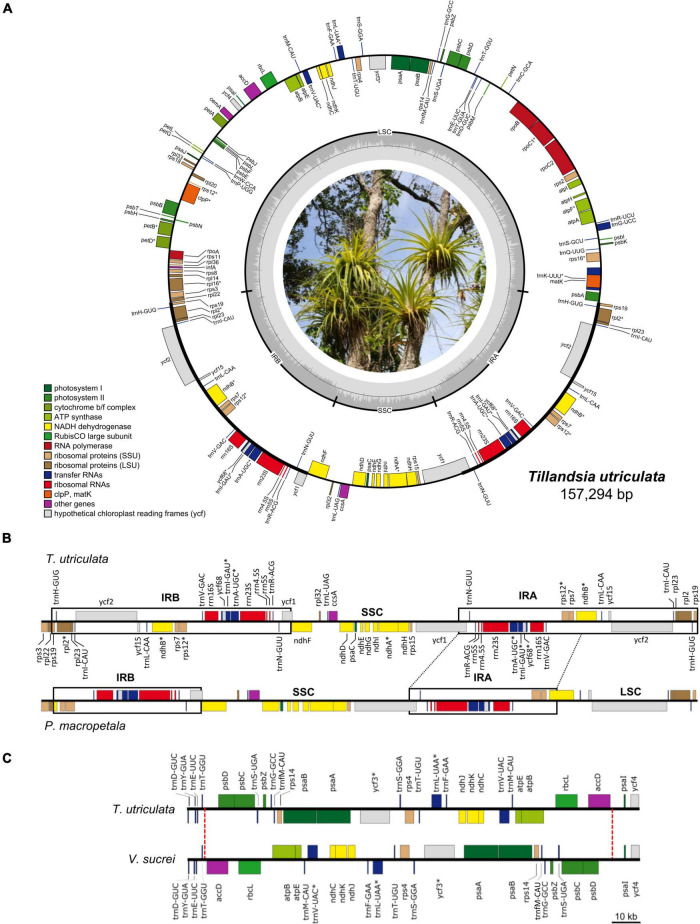
**(A)** Circular representation of the *Tillandsia utriculata* plastome (Photo credit: Juan Pablo Pinzón). Genes are color coded according to their functional category and GC content across the plastome is denoted by the innermost gray circle, with the 50% threshold of GC content marked by a thin gray line. Genes that are transcribed counter-clockwise and clockwise are displayed to the outside and the inside of the outer circle, respectively. Genes with introns are denoted by an asterisk (*). **(B)** Comparison of the IRB-SSC-IRA region between *T. utriculata* and *Pseudalcantarea macropetala*, the latter showing a marked IR reduction and associated LSC expansion. One partial (*ndh*B) and eight full (*rpl*2, *rpl*23, *rps*19, *trn*H-GUG, *trn*I-CAU, *trn*L-CAA, *ycf*15, and *ycf*2) genes are translocated from IR to the LSC region in *Pseudalcantarea* compared to other Tillandsioideae species. **(C)** Comparison of a portion of the LSC of *T. utriculata* and *Vriesea sucrei*. *Vriesea* species present a large inversion spanning from the partial *trn*T-*acc*D IGS to the partial *psb*D-*psa*I IGS (27,080 bp in *V. sucrei*). The red dashed lines mark the boundaries of the inversion.

The newly assembled Tillandsioideae plastomes have an average coverage of 92.22×, ranging from 19.93× in *Barfussia laxissima* to 185.46× in *T. utriculata*. The plastomes are quadripartite and consist of one LSC and one SSC that are separated by two copies of an IR (Figure 1A and Supplementary Material 4). The average size of these plastomes is 156,606.5 bp, with *Tillandsia gymnobotrya* having the largest plastome (159,182 bp) and *P. macropetala* the smallest (143,799 bp). Average size of the LSC is 86,478.1 bp and ranges from 84,649 bp in *Lemeltonia narthecioides* to 98,667 bp in *Pseudalcantarea viridiflora*, whereas the average size of the SSC is 18,138.54 bp, with *Tillandsia achyrostachys* and *Tillandsia ixioides* having the smallest (16,946 bp) and the largest (18,655 bp) SSC, respectively. The smallest IR regions belong to *P. macropetala* (13,492 bp) and the largest to *T. achyrostachys* (27,957 bp), with 25,994.97 bp being the average size (Table 1 and Supplementary Material 5).

**TABLE 1:** Minimum and maximum values for the studied Tillandsioideae plastome attributes.

	Size (bp)	% GC content	No. SSR/kb	No. tandem repeats/kb
Full plastome	143,799–159,182	37.20–37.46	0.17–0.35	0.37–0.54
LSC	84,649–98,667	35.02–35.84	0.26–0.53	0.45–0.76
SSC	16,946–18,655	31.17–31.67	0.16–0.48	0.11–0.54
IR	13,492–27,957	42.20–46.77	0–0.14	0.14–0.37
Coding regions	81,885–91,930	40.08–40.74	0.02–0.07	0.11–0.20
Non-coding regions	61,908–73,081	33.11–33.69	0.36–0.81	0.65–1.07

“SSR” stands for Simple Sequence Repeats and “kb” for kilobases.

Gene content is identical in all the studied Tillandsioideae plastomes and includes 115 unique coding genes, of which 81 are protein-coding genes, 30 tRNA genes, and four rRNA genes (Table 2 and Supplementary Material 4). Each IR contains eight complete (*rps*19, *rpl*2, *rpl*23, *ycf*2, *ycf*15, *ndh*B, *rps*7, and *ycf*68) and two partial (*rps*12 and *ycf*1) protein-coding genes, eight tRNA genes (*trn*H-GUG, *trn*I-CAU, *trn*L-CAA, *trn*V-GAC, *trn*I-GAU, *trn*A-UGC, *trn*R-ACG, and *trn*N-GUU) and the four rRNA genes, except for the two *Pseudalcantarea* species that have shorter IRs, thereby having the *rps*19, *trn*H-GUG, *rpl*2, *rpl*23, *trn*I-CAU, *ycf*2, *ycf*15, and *trn*L-CAA genes and a truncated *ndh*B in the LSC instead (Figure 1B).

**TABLE 2:** Genes present in the plastome of species of core Tillandsioideae.

Function	Group of genes	Gene names
Protein synthesis and DNA replication	Transfer RNAs	*trn*A-UGC*^a^* (×2), *trn*C-GCA, *trn*D-GUC, *trn*E-UUC, *trn*F-GAA, *trnf*M-CAU, *trn*G-GCC, *trn*G-UCC*^a^*, *trn*H-GUG (×2), *trn*I-CAU (×2), *trn*I-GAU*^a^* (×2), *trn*K-UUU*^a^*, *trn*L-CAA (×2), *trn*L-UAA*^a^*, *trn*L-UAG, *trn*M-CAU, *trn*N-GUU (×2), *trn*P-UGG, *trn*Q-UUG, *trn*R-ACG (×2), *trn*R-UCU, *trn*S-GCU, *trn*S-GGA, *trn*S-UGA, *trn*T-GGU, *trn*T-UGU, *trn*V-GAC (×2), *trn*V-UAC*^a^*, *trn*W-CCA, *trn*Y-GUA
	Ribosomal RNAs	*rrn*4.5S (×2), *rrn*5S (×2), *rrn*16S (×2), *rrn*23S (×2)
	Ribosomal protein small subunit	*rps*2, *rps*3, *rps*4, *rps*7 (×2), *rps*8, *rps*11, *rps*12*^a,c^* (×2), *rps*14, *rps*15, *rps*16*^a^*, *rps*18, *rps*19 (×2)
	Ribosomal protein large subunit	*rpl*2*^a^* (×2), *rpl*14, *rpl*16*^a^*, *rpl*20, *rpl*22, *rpl*23 (×2), *rpl*32, *rpl*33, *rpl*36
	Subunits of RNA polymerase	*rpo*A, *rpo*B, *rpo*C1*^a^*, *rpo*C2
Photosynthesis	Photosystem I	*psa*A, *psa*B, *psa*C, *psa*I, *psa*J
	Photosystem II	*psb*A, *psb*B, *psb*C, *psb*D, *psb*E, *psb*F, *psb*H, *psb*I, *psb*J, *psb*K, *psb*L, *psb*M, *psb*N, *psb*T, *psb*Z
	Cytochrome b/f complex	*pet*A, *pet*B*^a^*, *pet*D*^a^*, *pet*G, *pet*L, *pet*N
	ATP synthase	*atp*A, *atp*B, *atp*E, *atp*F*^a^*, *atp*H, *atp*I
	NADH-dehydrogenase	*ndh*A*^a^*, *ndh*B*^a^* (×2), *ndh*C, *ndh*D, *ndh*E, *ndh*F, *ndh*G, *ndh*H, *ndh*I, *ndh*J, *ndh*K
	Large subunit Rubisco	*rbc*L
Miscellaneous function	Translation initiation factor IF-1	*inf*A
	Acetyl-CoA carboxylase	*acc*D
	Cytochrome c biogenesis	*ccs*A
	Maturase	*mat*K
	ATP-dependent protease	*clp*P*^b^*
	Inner membrane protein	*cem*A
Protein transport through plastid membranes	Conserved hypothetical chloroplast ORF	*ycf*1 (×2), *ycf*2 (×2)
Potentially involved in the assembly of photosystem I	Conserved hypothetical chloroplast ORF	*ycf*3*^b^*, *ycf*4
Unknown function	Conserved hypothetical chloroplast ORF	*ycf*15 (×2), *ycf*68*^a^* (×2)

^a^Gene containing one intron.

^b^Gene containing two introns.

^c^Trans-spliced gene.

Gene order is highly conserved and changes in gene order were only found in the *Pseudalcantarea* and *Vriesea* representatives. In *Pseudalcantarea*, gene order change is caused by their shorter IR that spans from the partial *ycf*1 gene to the partial *ndh*B gene, instead of up to the *rps*19 gene as in all the remaining species, as a result, gene order in the LSC-IRb border differs from all other species (Figure 1B and Supplementary Material 4). Gene order changes in *Vriesea* derive from a large inversion of the region between the *acc*D and *psb*D genes, which inverts the order of a total of 24 genes relative to the remaining species (Figure 1C and Supplementary Material 4). An additional large inversion shared by the two *T. biflora* complex representatives was detected spanning the *acc*D gene and portion of the flanking IGS (Supplementary Material 4), however, since this inversion involves only one gene, the general gene order is not affected.

Coding regions, including protein-coding, tRNA and rRNA genes, accounted on average for 90,993.27 bp, ranging from 81,885 bp in *P. viridiflora* to 91,930 bp in *T. gymnobotrya*, whereas non-coding regions ranged from 61,908 to 73,081 bp in *P. macropetala* and *Lutheria splendens*, respectively (average 65,613.32 bp). The GC content of full plastomes ranged from 37.20% in *C. sessiliflora* to 37.46% in *Alcantarea odorata*, with an average of 37.34%. *Catopsis sessiliflora* presented the highest (0.35) number of SSRs per 1k bp in the complete plastome and *Wallisia cyanea* the lowest (0.17; average 0.25), whereas *T. ixioides* and *W. cyanea* had the highest (0.54) and lowest (0.37; average 0.45) number of tandem repeats per 1k bp in the complete plastome, respectively. Further details on the variation of the GC content, number of SSR and tandem repeats per 1k bp of the LSC, SSC, and the IR can be found the Table 1 and Supplementary Material 5.

### Phylogenetic relationships of the core Tillandsioideae

The alignment of the full plastomes resulted in a 168,047 bp long matrix. After the exclusion of one IR copy, small and large inversions, as well as ambiguously aligned regions for a total of 65,434 excluded sites, the final matrix used for phylogenetic inference comprised 102,613 aligned sites, including 54,560 bp corresponding to exons of protein-coding genes, 1,575 bp to exons of tRNA genes, 4,525 bp to rRNA genes, 12,774 bp to introns and 29,179 bp to IGS.

**FIGURE 2:**
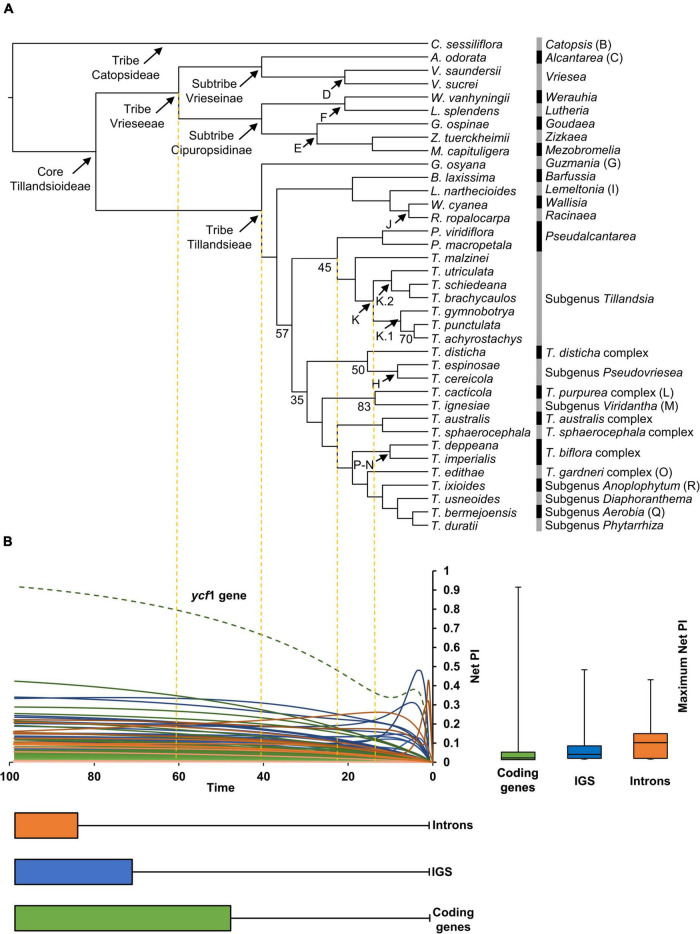
**(A)** Phylogenetic relationships obtained from the IQ-TREE ML analysis with branch lengths converted to ultrametric. Clade names and letters identifying genera, subgenera and species complexes follow Barfuss et al. (2016) and Granados Mendoza et al. (2017). Support values are shown below the branches for nodes with BS <85%. **(B)** Net phylogenetic informativeness (PI) profiles of plastid coding genes (green), intergenic spacers (blue), and introns (orange). The green dashed curve corresponds to the *ycf*1 gene, which surpassed by far the PI of any other marker. Distribution of loci’s maximum net PI values and time at which these values were reached is shown with quantiles 2 and 3 to the right and below the PI profiles, respectively. Whiskers denote maximum and minimum values. The arbitrary time scale of the PI profiles matches the one of the ultrametric tree in **(A)**.

Below is a description of the retrieved phylogenetic relationships that were all strongly supported (BS ≥90), except those stated below (Figure 2A). Our ML analysis retrieved tribes Vrieseeae and Tillandsieae as monophyletic. Within tribe Vrieseeae, two sister clades corresponding to subtribes Vrieseinae and Cipuropsidinae were recovered. Subtribe Vrieseinae includes the monophyletic *Vriesea* sister to *Alcantarea*, whereas subtribe Cipuropsidinae is further divided in two clades, the first of which includes *Lutheria* sister to *Werauhia* and the second including *Goudaea*, *Zizkaea*, and *Mezobromelia* as successive sisters. In Tribe Tillandsieae, *Guzmania* is recovered as sister to a clade including all the remaining species, which is further divided into two clades. The first clade includes *Barfussia*, *Lemeltonia*, *Wallisia*, and *Racinaea* as successive sisters and the second clade (BS = 57) is further divided in two lineages. The first of these lineages (BS = 45) includes the monophyletic *Pseudalcantarea* as sister to a monophyletic *Tillandsia* subgenus *Tillandsia*. Within this subgenus, *T. malzinei* is recovered as sister to clade K (*sensu* Barfuss et al., 2016), which is further divided into the clades K.1 and K.2 (*sensu* Granados Mendoza et al., 2017). Clade K.1 includes *T. gymnobotrya* as sister to the clade integrated by *Tillandsia punctulata* and *T. achyrostachys* (BS = 70), whereas clade K.2 includes *T. utriculata* as sister to the clade of *Tillandsia schiedeana* and *Tillandsia brachycaulos*. The second lineage includes the remaining *Tillandsia* species (BS = 35) divided in two clades. In the first of these clades (BS = 50) the *T. disticha* complex is sister to a monophyletic *T*. subgenus *Pseudovriesea*, whereas in the second clade the [*T. purpurea* complex + *T*. subgenus *Viridantha*] clade (BS = 83) is sister to a clade containing the [*T. australis* + *T. sphaerocephala* complexes] clade as sister to a lineage including [*T. biflora* + *T. gardneri* complexes] clade, and subgenera *Anoplophytum*, *Diaphoranthema*, *Aerobia*, and *Phytarrhiza* as successive sisters (Figure 2A).

### Phylogenetic utility of plastid markers and data categories

In general, PI profiles showed curves that steadily increased reaching their maximum net PI toward time 100. Some IGS and introns showed steep increases corresponding to shallow divergences followed by more gradual decreases toward time 100. A few introns and the *ycf*1 gene presented spikes toward time 0, corresponding to sites with unusually high substitution rates compared to the remaining sites in the alignments (Figure 2B). Median of maximum net PI was higher for introns, followed by IGS and coding genes (9.1 × 10^–2^, 2.8 × 10^–2^, and 9.4 × 10^–3^, respectively), with the *ycf*1 gene as outlier with 0.92 of maximum net PI. Twenty-two coding regions, including six exons of protein-coding genes, 14 exons of tRNA genes and two rRNA genes, had zero maximum net PI. Among informative coding regions, net PI ranged from 3.42 × 10^–46^ in the *pet*G gene to 0.92 in the *ycf*1 gene. The *psb*F-*psb*E, *rpl*23-*rpl*2, and *rrn*4.5S-*rrn*23S IGS had zero maximum net PI, while maximum net PI of informative IGS ranged from 5.27 × 10^–129^ in the *trn*L-*ccs*A IGS to 0.48 in the *psa*C-*ndh*E IGS. Only the second intron of the *trn*I-GAU gene had zero maximum net PI. Maximum net PI of the remaining introns ranged from 2.92 × 10^–3^ in the *ycf*68 intron to 0.42 in the *rpl*16 intron. Most loci reached their maximum net PI toward the root, with a median value of 99 for the three data categories. When considering the three data categories together, the 15 most informative loci are: *ycf*1 gene, *psa*C-*ndh*E IGS, *rpl*16 intron, *rpo*C2 gene, *psb*E-*pet*L IGS, *trn*S-*trn*G IGS, *clp*P intron 1, *rpl*32-*trn*L IGS, *ndh*F gene, *atp*F intron, *mat*K gene, *pet*N-*psb*M IGS, *rpo*B-*trn*C IGS, *trn*T-*psb*D IGS, and *rpo*B gene (Figure 2B and Supplementary Material 3).

### Ancestral state reconstruction and detection of rate shifts of plastome attributes

The crown node of core Tillandsioideae was reconstructed with a plastome size of 157,138.08 bp (95% CI = 152,990.92–161,285.24 bp), and sizes of LSC, SSC and IR regions of 86,271.32 bp (95% CI = 82,517.65–90,024.99 bp), 18,228.11 bp (95% CI = 17,634.68–18,821.55 bp), and 26,319.32 bp (95% CI = 22,392.93–30,245.70 bp), respectively. A total GC content of 37.34% (95% CI = 37.26–37.43%) was reconstructed for the root node, whereas reconstructed total number of SSR and tandem repeats per 1 kb were 0.26 (95% CI = 0.21–0.30) and 0.45 (95% CI = 0.37–0.53), respectively. Ancestral state reconstruction, variance, and 95% confidence intervals for the 20 studied plastome attributes for each node of the Tillandsioideae tree can be found in the Supplementary Material 6.

**FIGURE 3:**
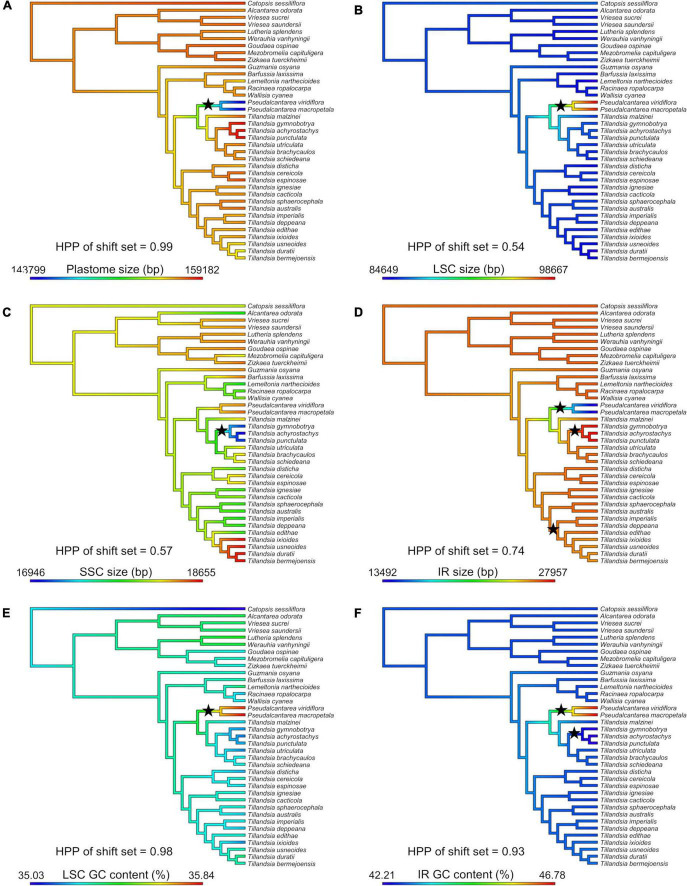
Evolution of six plastome attributes where evolutionary rate shift sets were detected by BAMM with PP ≥0.5. Ancestral state reconstruction was inferred under a Maximum Likelihood (ML) framework and mapped on the Tillandsioideae phylogeny. **(A)** Plastome size. **(B)** LSC size. **(C)** SSC size. **(D)** IR size. **(E)** LSC GC content. **(F)** IR GC content. LSC, large single copy; SSC, small single copy; IR, inverted repeat; bp, base pairs; HPP, highest posterior probability. Black stars indicate the location of detected evolutionary rate shifts.

Shifts in evolutionary rate were detected in seven out of 20 attributes (Figure 3). A single evolutionary rate shift was detected for several attributes, including plastome size (PP = 0.99; Figure 3A), LSC size (PP = 0.54; Figure 3B), GC content of the LSC (PP = 0.98; Figure 3E; the three attributes shifting at the *Pseudalcantarea* clade), SSC size (PP = 0.57; Figure 3C; at the K.1 clade), and number of tandem repeats in the SSC (PP = 0.18; not illustrated; at Tribe Tillandsieae clade). GC content of the IR had two evolutionary rate shifts (PP = 0.93; Figure 3F) at the *Pseudalcantarea* and K.1 clades, whereas IR size had three evolutionary rate shifts (PP = 0.74; Figure 3D) at the *Pseudalcantarea* and K.1 clades, as well as a clade including *T. biflora* and *T. gardneri* complexes, and the subgenera *Anoplophytum*, *Diaphoranthema*, *Aerobia*, and *Phytarrhiza*.

## Discussion

### New lineage-specific plastome rearrangements support recently adopted taxonomic groups

Our study results in 34 new, complete plastomes for Tillandsioideae species (plastomes for three of the herein sampled species were already available in Genbank), significantly enriching Bromeliaceae genomic resources (Supplementary Material 4, 5A). Compared to the previously published Tillandsioideae plastomes, nine of the herein studied species have smaller plastomes, the lower bound being the plastomes of the two *Pseudalcantarea* species (143,799 and 144,169 bp). Range of the previously reported size for the LSC, SSC, and IR in Tillandsioideae was similarly expanded by our results, being remarkable the considerably smaller IR regions (13,544 to 13,492 bp) and larger LSC region (98,341 and 98,667 bp) of the two *Pseudalcantarea* species (Supplementary Material 5A). Variation of plastome size in land plants has been attributed to several reasons, including expansions and contractions of the IR or loss of one of the copies, gene loss or pseudogenization (frequently involving the *ndh* gene group), intron loss, IGS size variation, and differences in the abundance of repetitive sequences (Ruhlman et al., 2015; Mower and Vickrey, 2018). As discussed below, expansions and contractions of the IR seem to play a major role for the observed plastome size differences in Tillandsioideae, but gene pseudogenization, IGS size variation, and differences in the abundance of short repetitive sequences also play a role, although to a lesser extent.

To our knowledge, besides the two *Pseudalcantarea* species herein studied, there is no other Bromeliaceae report with this noticeable IR reduction and associated LSC expansion. In fact, the IR size of the *Pseudalcantarea* species is smaller than the typical range reported for land plants (15–30 kb; Zhu et al., 2016). Despite these structural differences, gene content is identical across the analyzed Tillandsioideae plastomes (Table 2 and Supplementary Material 4) since the genes that are absent in the IR region of the *Pseudalcantarea* plastomes (but present in the IR of all other Tillandsioideae species) are instead translocated into the LSC region and thus expanding its size (Figure 1B). Large IR contractions (>10,000 bp) are less frequent than IR expansions in autotrophic vascular plants (Mower and Vickrey, 2018) and, to our knowledge and when excluding complete losses of one IR, large IR contractions are only known from Austrobaileyales (Hansen et al., 2007; Gruenstaeudl et al., 2017). As currently circumscribed, *Pseudalcantarea* includes the two species here sampled plus *Pseudalcantarea grandis*. Confirming the monophyly of *Pseudalcantarea* (as currently circumscribed) and assessing whether *P. grandis* shares the IR reduction and associated LSC expansion is central for recognizing this feature as a molecular synapomorphy for this lineage.

Overall gene content and order are highly conserved across Bromeliaceae, a single difference was observed between our gene annotations and those of previous studies. The *ycf*68 gene has been previously annotated only for the MD-2 *A. comosus* commercial hybrid (Redwan et al., 2015) and *A. comosus* var. *erectifolius* (Liu et al., 2022), but not in other Bromeliaceae. However, we found that this gene corresponds to an open reading frame in all Tillandsioideae studied species, embedded in the *trn*I-GAU intron, and is composed of two exons and an intron (Figure 1A and Supplementary Material 4), as first annotated by Redwan et al. (2015).

Three potential pseudogenes were identified, including the *ycf*1 and *ycf*15 genes, in all studied species, and the *ndh*B gene only in *Pseudalcantarea*. The *ycf*1 gene is truncated at the IRb-SSC border and has also been annotated as a pseudogene in *A. comosus*, *T. espinosae*, *T. landbeckii*, *T. malzinei*, *T. purpurea*, and *T. usneoides* (Nashima et al., 2015; Poczai and Hyvönen, 2017; Chávez-Galarza et al., 2021; Möbus et al., 2021), as well as other monocots such as *Typha latifolia* (Poales), *Zingiber spectabile*, and *Musa textilis* (Zingiberales; Poczai and Hyvönen, 2017). The second pseudogene, *ycf*15, presented premature stop codons, as found in other Bromeliaceae (Nashima et al., 2015; Poczai and Hyvönen, 2017; Möbus et al., 2021). However, as discussed by Poczai and Hyvönen (2017) and thoroughly studied by Shi C. et al. (2013), even from intact *ycf*15 gene copies, known from several asterids, *Magnolia* (Magnoliales), *Piper* (Piperales), and *Camellia* (Ericales), it has not been possible to confirm if this gene is able to encode a protein.

In *Pseudalcantarea* the *ndh*B gene is truncated at the LSC-IRb border. As mentioned above, the *ndh*B is one of six core protein-coding genes of the angiosperms’ IR. Pseudogenization of one of the two generally present copies of the *ndh*B gene in species of *Pseudalcantarea* could be influencing this gene’s dosage and affecting its overall transcription levels. Even in the highly contracted IR of *Illicium* and *Schisandra* (Austrobaileyales), the *ndh*B gene remains intact (Gruenstaeudl et al., 2017). Experiments with tobacco plants with fully inactivated *ndh*B genes showed enhanced growth retardation and decline in photosynthesis under humidity stress (Horváth et al., 2000). *Pseudalcantarea* is composed of three mesomorphic, bat-pollinated species restricted to humid montane forest of Mexico (Aguilar-Rodríguez et al., 2014, 2019, Göttlinger et al., 2019). The reduced *ndh*B gene dosage of *Pseudalcantarea* species might explain the restriction of this lineage to highly humid environments and be related to its low taxic diversity, since its physiological constrains might have impeded the dispersion of this lineage to less suitable environments.

Among Bromeliaceae, *Vriesea* is the first genus reported to have an inversion of significant size (27,080–27,120 bp), which is located at the LSC and spans from the *acc*D gene to the *psb*D gene, including parts of the two flanking IGS (Figure 1C and Supplementary Material 4). The breaking point toward the *acc*D gene is near the *trn*T-GGU gene, whereas the breaking point toward the *psb*D gene is close to a SSR and a tandem repeat, which agrees with previous observations that breaking points of inversions are frequently flanked by tRNA genes and shorth repeat sequences (Wicke et al., 2011). Previous studies focused on the systematics of this genus (Machado et al., 2020) did not report this inversion, most probably because they retrieved only a partial plastome for *Vriesea marceloi* and, for other species, the authors exclusively extracted individual genes. Wu et al. (2022) also did not report this inversion for the *V.* × *poelmanii* hybrid, although after analyzing their sequence we can confirm that the inversion is present. Exploring the presence of this large inversion in other species of *Vriesea*, as well as in species of the closely related genus *Stigmatodon* (Machado et al., 2020), can help determine whether this inversion represents a molecular synapomorphy for *Vriesea* only or for the *Vriesea* + *Stigmatodon* lineage.

We found that the *acc*D gene and part of each of its flanking IGS is inverted in the studied *T. biflora* complex representatives relative to all other studied Tillandsioideae plastomes (Supplementary Material 4). Further comparison to other previously published Bromeliaceae plastomes revealed that the inversion seems to be restricted to the *T. biflora* species complex. Harris et al. (2013) studied the evolution of the *acc*D gene in Poales and found that this gene is present in *Tillandsia secunda* (*T*. subgenus *Tillandsia*), as well as in representatives of Typhaceae, Cyperaceae, Juncaceae, Eriocaulaceae, Xyridaceae, and Flagellariaceae. However, Harris et al. (2013) also found that the *acc*D has been lost in representatives of Restionaceae, Joinvilleaceae, and Poaceae, and pseudogenized in Poaceae, suggesting a certain degree of lability of the gene in this order. Considering that the *T. biflora* complex is a highly speciose lineage including over 136 species (Barfuss et al., 2016), a more representative taxon sampling within this species complex would be necessary to confirm or refute this inversion as a synapomorphy for the group.

Three plastomes from species that were already available in GenBank, including *T. espinosae*, *T. malzinei* (Möbus et al., 2021), and *T. usneoides* (Poczai and Hyvönen, 2017), were here newly sequenced and assembled. A few differences were found compared to the previously published plastomes of *T. espinosae* (7 bp), and *T. malzinei* (85 bp). However, over 5,564 sites were different between the two plastome sequences of *T. usneoides*. These numerous differences are evenly distributed across the plastomes and could be attributed to the species’ ample geographic distribution, which essentially equals the distribution range of the genus *Tillandsia* (south of United States to central Argentina), potentially promoting increased infraspecific genetic variability.

### Performance of full plastomes in resolving core Tillandsioideae phylogeny

Analysis of complete plastome sequences contributed to the resolution of most, but not all, recalcitrant relationships within core Tillandsioideae. Our results are in general consistent with the phylogenetic relationships recovered by previous studies using plastid data (Barfuss et al., 2016; Machado et al., 2020; Loiseau et al., 2021), albeit in discordance with the phylogenetic relationships retrieved by Loiseau et al. (2021) based on nuclear data, where subtribe Cipuropsidinae (tribe Vrieseeae) is recovered as sister to tribe Tillandsieae (vs. sister to subtribe Vrieseinae), and *Werauhia* is retrieved as sister to the remaining genera of subtribe Cipuropsidinae (vs. sister to *Lutheria*). Genus level phylogenetic relationships obtained by Yardeni et al. (2022) from the analysis of their nuclear dataset are mostly in agreement with our results, except for the position of *Racinaea* which is recovered nested within *Tillandsia* by these authors. Despite the differences in taxon samplings across studies, the consistently retrieved strongly supported phylogenetic discordance between plastid and nuclear data sources suggest that evolutionary processes, such as hybridization or lineage sorting, could have shaped the evolution of the core Tillandsioideae. This is in line with the results of Loiseau et al. (2021), who documented widespread intra and intergeneric hybridization, both within and among tribes of Tillandsioideae, which might at least partially explain the discordance between plastid and nuclear data sources. Future studies using increased taxon samplings and including both uniparentally and biparentally inherited molecular sources of data could address the potential sources of the observed phylogenetic discordance.

Below is a discussion of the herein retrieved phylogenetic relationships relative to the study of Barfuss et al. (2016), which remains to date the more comprehensive phylogenetic study of the Tillandsioideae. However, it is important to notice that our taxon sampling is considerably limited, albeit representative, compared with Barfuss et al. (2016). All genera, subgenera and species complexes including more than one sampled species in the present study were recovered as monophyletic, except *Tillandsia*, as *T*. subgenus *Tillandsia* was recovered sister to genus *Pseudalcantarea* (BS = 45). The phylogenetic position of *Pseudalcantarea* within tribe Tillandsieae remains unsolved as backbone relationships were weakly supported by our and previous studies (e.g., Barfuss et al., 2016). Future studies providing higher resolution for these recalcitrant relationships could confirm or refute Barfuss et al.’s (2016) proposal of keeping *Pseudalcantarea* as a segregated genus of *Tillandsia*.

Some previously identified, but weakly supported groups by Barfuss et al. (2016) received increased statistical support in our study, including the clade [*Zizkaea* + *Mezobromelia*] (BS = 93 vs. 61), the clade [*Barfussia* + *Lemeltonia* + *Wallisia* + *Racinaea*] (BS = 96 vs. 62), the clade comprising *T. biflora* and *T. gardneri* species complexes plus subgenera *Anoplophytum*, *Diaphoranthema*, *Aerobia*, and *Phytarrhiza* (BS = 100 vs. 58), and the clade of *T. biflora* species complex (BS = 100 vs. 65). Although some backbone relationships within genus *Tillandsia* remain weakly supported in our analysis, our study recovered subgenera *Anoplophytum* and *Diaphoranthema* as successive sisters of the clade including subgenera *Aerobia* and *Phytarrhiza* as highly supported (BS = 100), which were not resolved in Barfuss et al. (2016; Figure 2A). The overall congruence of our results and those previously published based on plastid information, along with the increased statistical support for previously identified weakly supported clades, suggest full plastomes as a valuable source of data for phylogenetic studies in Tillandsioideae. Future studies using full plastomes and denser taxon sampling will test if the herein recovered highly supported clades hold true when the taxon sampling is increased. Resolution for recalcitrant phylogenetic relationships within tribe Tillandsieae could benefit from the inclusion of NGS based nuclear data, however, as already pointed out by Loiseau et al. (2021), phylogenomic discordance between plastid and nuclear data is expected to emerge.

### Old and new highly informative plastid loci

We explored the PI of all loci included in our final plastid matrix, which comprised all coding regions, introns and IGS (Figure 2B and Supplementary Material 3), except for those placed within the large inversion detected in the LSC of *Vriesea*, which also contains the smaller inversion detected for the *T. biflora* species complex. Our decision to exclude this large inversion, as well as other detected minor inversions, was because sites within these inversions are not homologous across the herein studied species. As a result, we excluded 15 protein-coding genes, eight tRNA genes, and 22 IGS prior to the phylogenetic analysis. However, other studies not including *Vriesea* or *T. biflora* species or only including these two taxa separately, could make use of the herein excluded plastid loci and potentially improve phylogenetic resolution. Four of the excluded loci, that are present within the large inversion (i.e., *rbc*L gene, *atp*B-*rbc*L IGS, *trn*L-*trn*F IGS, and *trn*L intron), have been employed in a previous phylogenetic study including *Vriesea* species and other Tillandsioideae (Barfuss et al., 2005). However, these markers were sequenced with Sanger, hence the non-homology of these regions could not have been detected (assuming the inversion is shared across *Vriesea* species).

Several other Sanger sequenced markers have been employed in previous molecular phylogenetic studies of the Tillandsioideae, including the *ndh*F and *ycf*1 genes, the *rpl*32-*trn*L and *trn*K-*rps*16 IGS, the *rps*16 intron, and the *trn*K-*mat*K-*trn*K, *rpo*B-*trn*C-*pet*N, *trn*D-*trn*Y-*trn*E-*trn*T, and *trn*H-*rps*19-*psb*A regions (Terry et al., 1997; Barfuss et al., 2005, 2016; Granados Mendoza, 2008; Chew et al., 2010; Versieux et al., 2012; Donadío, 2013; Castello et al., 2016; Pinzón et al., 2016; Leme et al., 2017; Kessous et al., 2020; Donadío et al., in press). Among these markers, the *ycf*1 gene far exceeds the PI of any other analyzed loci (Figure 2B and Supplementary Material 3). The high phylogenetic utility of the *ycf*1 gene has been widely recognized in several vascular plant lineages (e.g., Chase et al., 2009; Gernandt et al., 2009; Neubig et al., 2009, 2012; Parks et al., 2009; Drew and Sytsma, 2013; Shi S. et al., 2013; Arévalo et al., 2015), generally surpassing the utility of other plastid markers and, in some cases, nuclear loci (Granados Mendoza et al., 2020).

Other previously used markers in Tillandsioideae were here recovered as highly informative, including the *rpo*B-*trn*C-*pet*N region, *mat*K-*trn*K region, *rpl*32-*trn*L IGS and *ndh*F gene, which were recovered among the 15 most informative loci. The *trn*D-*trn*Y-*trn*E-*trn*T region, *trn*K-*rps*16 IGS, *trn*H-*rps*19-*psb*A region, and *rps*16 intron rank 22nd, 25th, 32nd, and 35th, out of 217 analyzed loci. Our results not only suggest that plastid marker selection in Tillandsioideae has been in general appropriate, but also identify other loci, i.e., *psa*C-*ndh*E IGS, *rpl*16 intron, *rpo*C2 gene, *psb*E-*pet*L IGS, *trn*S-*trn*G IGS, and *clp*P intron 1, as additional promising markers for future Sanger based studies of the Tillandsioideae (Supplementary Material 3), some of them already known for their utility in angiosperm phylogenetic studies (e.g., Shaw et al., 2007).

Overall, PI of non-coding regions (i.e., introns and IGS) was higher than that of coding genes (Figure 2B), which is in agreement with a general trend since coding regions are known to have low rates of nucleotide substitution (Clegg et al., 1994). However, it is noteworthy that the *trn*I-GAU intron and the *psb*F-*psb*E, *rpl*23-*rpl*2, and *rrn*4.5S-*rrn*23S IGS had PI close to zero (Supplementary Material 3). The *trn*I-GAU gene and the four rRNA genes are part of a highly conserved core gene set shared in plant and algae IRs (Zhu et al., 2016). As a result, the non-coding *trn*I-GAU intron and *rrn*4.5S-*rrn*23S IGS could also be affected by the reduced substitution rates. Similarly, the *rpl*23 and *rpl*2 genes form part of a set of protein-coding genes (also including the *rps*12-3′, *rps*7, *ndh*B, and *ycf*2) that were ancestrally present in the angiosperms’ IR and that are known to present low sequence divergence (Zhu et al., 2016), suggesting that low substitution rates can also affect their IGS. The fact that most loci reached their maximum net PI toward the root (median = 0.99), coincides with the lack of statistical support at shallower nodes within tribe Tillandsieae (Figure 2 and Supplementary Material 3). Also, the trend followed by most of the analyzed plastid loci of reaching their maximum net PI toward the root indicates that plastid markers could be more informative for deeper phylogenetic divergences, for instance, for informing phylogenetic relationships among Bromeliaceae subfamilies. The lack of statistical support for shallower divergences retrieved by the analyses of full plastomes points toward the need of future studies that analyze more variable molecular markers such as low- or single-copy nuclear genes.

### Evolution of plastome attributes and traits’ rates shifts

Ten evolutionary shifts in the rate of evolution were detected in seven plastome attributes, corresponding to seven evolutionary rate shift sets. The following discussion focuses on evolutionary rate shifts sets with posterior probability ≥0.50 (Figure 3). The node subtending the two *Pseudalcantarea* representatives allocated 5 of the 10 shifts, including plastome, LSC and IR sizes, as well as GC content of the LSC and IR regions. As mentioned above, *Pseudalcantarea* plastomes showed an important IR contraction and associated LSC expansion, with one partial (*ndh*B) and eight full copy (*rpl*2, *rpl*23, *rps*19, *trn*H-GUG, *trn*I-CAU, *trn*L-CAA, *ycf*15, and *ycf*2) genes translocated from IR to the LSC region. Most translocated genes in *Pseudalcantarea* are involved in protein synthesis, DNA replication, protein transport through plastid membranes or have an undefined function (Table 2). The only translocated gene involved in photosynthesis is the *ndh*B gene, although, it was only partially translocated into the LSC region. IRs have been proposed as stabilizing regions and are the most conserved regions of the plastomes, with reduced rates of synonymous nucleotide substitutions and evolving up to 3.7 times slower than the single copy regions. Conversely, the LSC is thought to evolve more rapidly and to be the least conserved of the plastome regions (Wolfe et al., 1987; Ravi et al., 2008; Zhu et al., 2016). Perry and Wolfe (2002) reported for IR-lacking legumes that the translocation of “ancestral” IR genes into the single copy region derived on an increment on substitution rates of the genes, and this trend was confirmed by a recent and ample study on vascular plants (Zhu et al., 2016). Future studies could address if substitution rate and, potentially GC content, of the genes translocated into the LSC in *Pseudalcantarea* also experience increased substitution rates compared to those present in the IR regions of all other Tillandsioideae species. Notably, as found by Gruenstaeudl et al. (2017) in Austrobaileyales, the border of the contracted IR and the LSC is at the *ndh*B gene, the same as in *Pseudalcantarea*, although the gene copy is not truncated in Austrobaileyales. Future transcriptome studies might help elucidate the effects on expression of the translocated genes in *Pseudalcantarea* as gene dosage might be altered compared to other Tillandsioideae lineages. Furthermore, a denser taxon sampling could help addressing if the detected evolutionary plastome traits’ rate shifts of *Pseudalcantarea* are associated to diversification rate shifts (e.g., low speciation and/or high extinction rates) of this lineage.

Three additional shifts were allocated at the K.1 clade, which is one of the main clades of *Tillandsia* subgenus *Tillandsia*, including size of the SSC and IR and GC content of the IR. The three sampled species of clade K.1 (i.e., *T. gymnobotrya*, *T. punctulata*, and *T. achyrostachys*) have expanded IRs at the IR-SSC borders resulting in the translocation of a portion of the *ycf*1 gene/pseudogene from the SSC to the IR and the associated SSC region reduction. Translocations from single copy regions into the IR have already been reported in Bromeliaceae for the genera *Fascicularia* and *Ochagavia* by Paule et al. (2020), however, in their case translocation occurred from the LSC into the IR and not from the SSC as in clade K.1. Paule et al. (2020) findings and our results suggest that IR expansions and contractions could have played a key role in plastome evolution in Bromeliaceae. The study of yet unexplored lineages and species could reveal additional cases of gene translocation from and to the IR.

Li et al. (2016) found that genes translocated from the single copy regions to the IR regions in ferns showed a two- to three-fold deceleration in their substitution rates and that the GC content of the third codon position and of the non-coding regions was significantly increased. This pattern was also confirmed for other vascular plant lineages by Zhu et al. (2016). However, in clade K.1 the translocation is associated to a decrement in the GC content of the IR (Supplementary Material 6), rather than an increment as in ferns. A possible explanation for this could be the differences in time after these shifts occurred. The two more closely related fern lineages involved in the translocation, Polypodiales and Gleicheniales, diverged at least 275 mya (Li et al., 2016), allowing enough time for achieving the “normal” behavior of the IR after the translocation. To our knowledge, there is no study specifically dating the origin and diversification of clade K.1. However, a crown age of less than 10 mya has been proposed for Tillandsioideae (Givnish et al., 2014) and of 6.6 mya for *Tillandsia* subgenus *Tillandsia* (Möbus et al., 2021), suggesting that clade K.1 is an extremely young lineage and that the copy-dependent repair phenomenon maintaining the generally observed IR GC content bias could still be at an early stage. A previous study reported that the clade K.1 is composed of at least 35 spp. distributed in North and Central America (Granados Mendoza et al., 2017), however, this clade is thought to be highly diverse. Future studies could test if the detected evolutionary rate shifts of plastome attributes could be associated to increased diversification rates of clade K.1.

An additional evolutionary rate shift was detected for the size of the IR located at a diverse clade including the *T. biflora* complex (136 spp.), *T. gardneri* complex (17 spp.), and subgenera *Anoplophytum* (33 spp.), *Diaphoranthema* (30 spp.), *Aerobia* (50 spp.) and *Phytarrhiza* (11 spp.; Barfuss et al., 2016) as successive sisters. The three representatives of the *T. biflora* and *T. gardneri* complexes present the typical IR-SSC border shared among most Tillandsioideae species, however, all representatives of the subgenera *Anoplophytum*, *Diaphoranthema*, *Aerobia*, and *Phytarrhiza* have experienced an IR contraction at the border with the SSC. As explained above for the K.1 clade, this case of IR reduction also involves a portion of the *ycf*1 gene and potential pseudogene suggesting this as a labile region promoting IR expansions and contractions.

## Conclusion

Analysis of full plastomes provided increased statistical support for several previously identified, weakly supported clades within core Tillandsioideae. Strongly supported relationships herein obtained agreed in general with previously published phylogenetic contexts based on plastid data, but phylogenetic discordance was detected relative to previous nuclear based studies. The use of nuclear NGS data could aid in resolving recalcitrant relationships of the group, however, phylogenetic discordance is expected to emerge due to widespread intra- and intergeneric hybridization in the subfamily. Overall, selection of plastid markers for phylogenetic inference of the core Tillandsioideae has been adequate, yet new highly informative plastid loci are here proposed for Sanger based studies. Tillandsioideae plastome structure is overall conserved, but new plastome rearrangements were discovered and found to support recently defined taxonomic groups. Future studies including expanded taxon samplings could confirm or refute these structural changes as molecular synapomorphies for the involved lineages. Furthermore, as discovered in this study, new plastome structural changes may arise from yet unexplored taxa. Here, IR expansions and contractions, as well as large inversions were found to play a role on the evolution of core Tillandsioideae plastomes. Studies with a wider taxonomic scope could help address how frequent these structural changes in Bromeliaceae are. Evolutionary rate shifts of plastome attributes were found associated to lineages with both high and low diversity. Future studies could formally test if the herein detected evolutionary rate shifts of plastome attributes are associated to significant changes in diversification rate across the core Tillandsioideae.

## Data availability statement

The data presented in the study are deposited in the National Center for Biotechnology Information (https://www.ncbi.nlm.nih.gov/) repository under the accession numbers ON398129–ON398163 and BK061352–BK061353.

## Author contributions

CGM, SV-P, DD, and SW conceived and designed the study. CGM, SV-P, DD, SW, GS, AR, EG, IR-M, and SD designed the taxon sampling and collected or provided the samples. SV-P, DD, and CGM performed the laboratory work. SV-P, DD, MJ, and CGM performed the bioinformatic process. CGM, SV-P, DD, MJ, and RH-G designed and performed the analyses. CGM, SV-P, DD, MJ, and AR drafted the manuscript. SW, RH-G, GS, SM, EG, IR-M, and SD proofread and approved the final manuscript. All authors contributed to the article and approved the submitted version.

## Footnotes

1
https://arborbiosci.com/

2
https://arborbiosci.com/mybaits-v5-chemistry/

3
https://digitalinsights.qiagen.com

4
https://www.geneious.com/

5
http://lowelab.ucsc.edu/tRNAscan-SE/

6
http://iqtree.cibiv.univie.ac.at/

7
https://github.com/faircloth-lab/tapir

8
http://bamm-project.org/quickstart.html#phenotypic-evolution

## Supplementary material

Supplementary Material 1 Taxon sampling and voucher information including collector, and collection number (herbarium code as in http://sweetgum.nybg.org/science/ih/).

Supplementary Material 2 Database of 47 complete plant plastomes used to mine contigs of possible plastome origin in our first *de novo* assembly strategy.

Supplementary Material 3 **(A)** Maximum net phylogenetic informativeness (PI) and the time at which it was reached for each analyzed locus. Loci were sorted by the net PI from high to low informativeness. Loci highlighted in green have been used in previous phylogenetic studies of Tillandsioideae. Net PI of regions was calculated by adding the PI values of each individual locus involved. **(B)** Raw phylogenetic informativeness profile data for each analyzed partition.

Supplementary Material 4 Circular representation of the plastome of all studied species. Genes are color coded according to their functional category and GC content across the plastome is denoted by the innermost gray circle, with the 50% threshold of GC content marked by a thin gray line. Genes that are transcribed counter-clockwise and clockwise are displayed to the outside and the inside of the outer circle, respectively.

Supplementary Material 5 **(A)** Plastome, LSC, SSC, and IR size comparison between previously published and herein generated plastomes. **(B)** Raw values for the 20 studied plastome attributes, including maximum and minimum values, for the complete taxon sampling.

Supplementary Material 6 Reconstructed ancestral state node values, including their variance, as well as lower and upper 95% CI, for the 20 studied plastome attributes on the Tillandsioideae phylogeny.

Supplementary Material 7 BAMM results for six plastome attributes where evolutionary rate shift sets were detected with PP ≥ 0.5. Branch colors reflect the rate of the trait evolution.
