# Supplementary figures and images for: New plastome structural rearrangements discovered in core Tillandsioideae (Bromeliaceae) support recently adopted taxonomy
Source: Front Plant Sci. 2022 Aug 1;13:924922. doi: 10.3389/fpls.2022.924922 (PMC9378858; doi:10.3389/fpls.2022.924922)

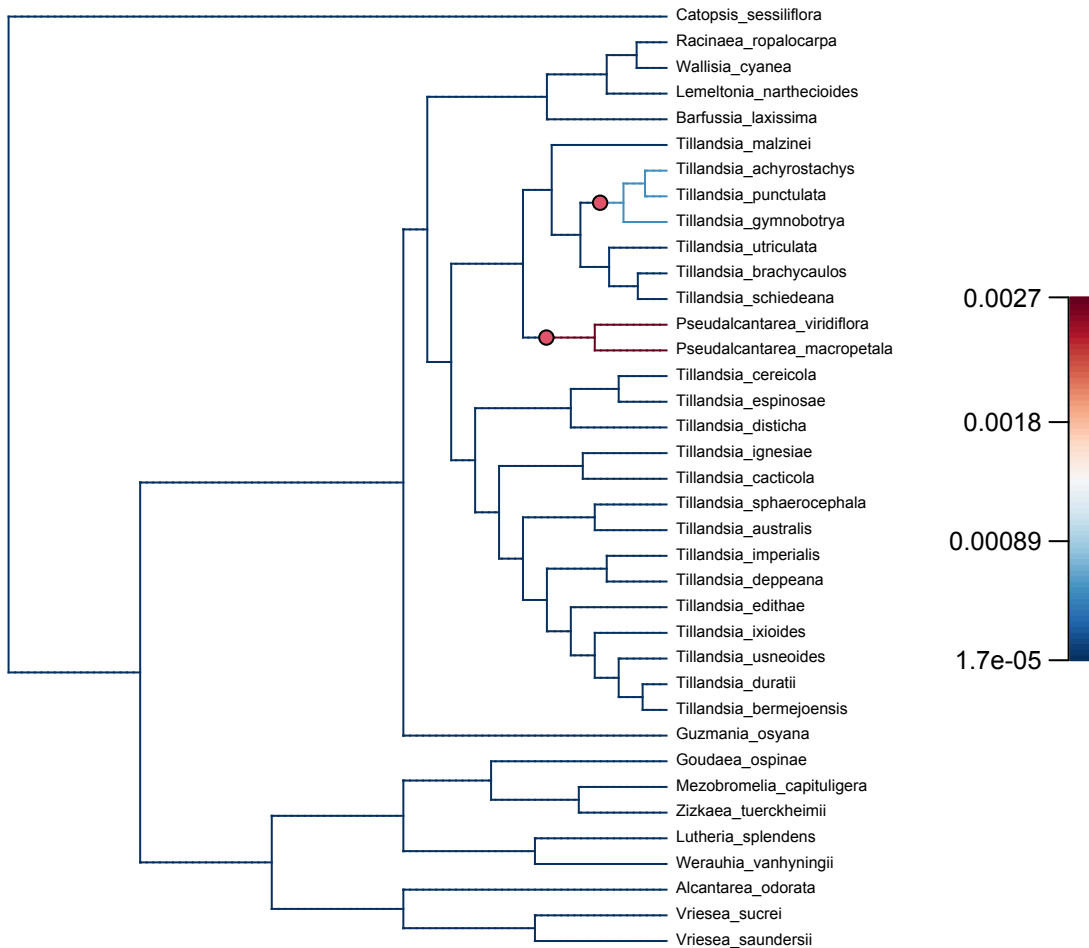

Supplement: Supplementary Material 7 — BAMM results for six plastome attributes where evolutionary rate shift sets were detected with PP ≥ 0.5. Branch colors reflect the rate of the trait evolution. [file Data_Sheet_3.ZIP › Supplementary_Material_S7/IR_GC_content.pdf]

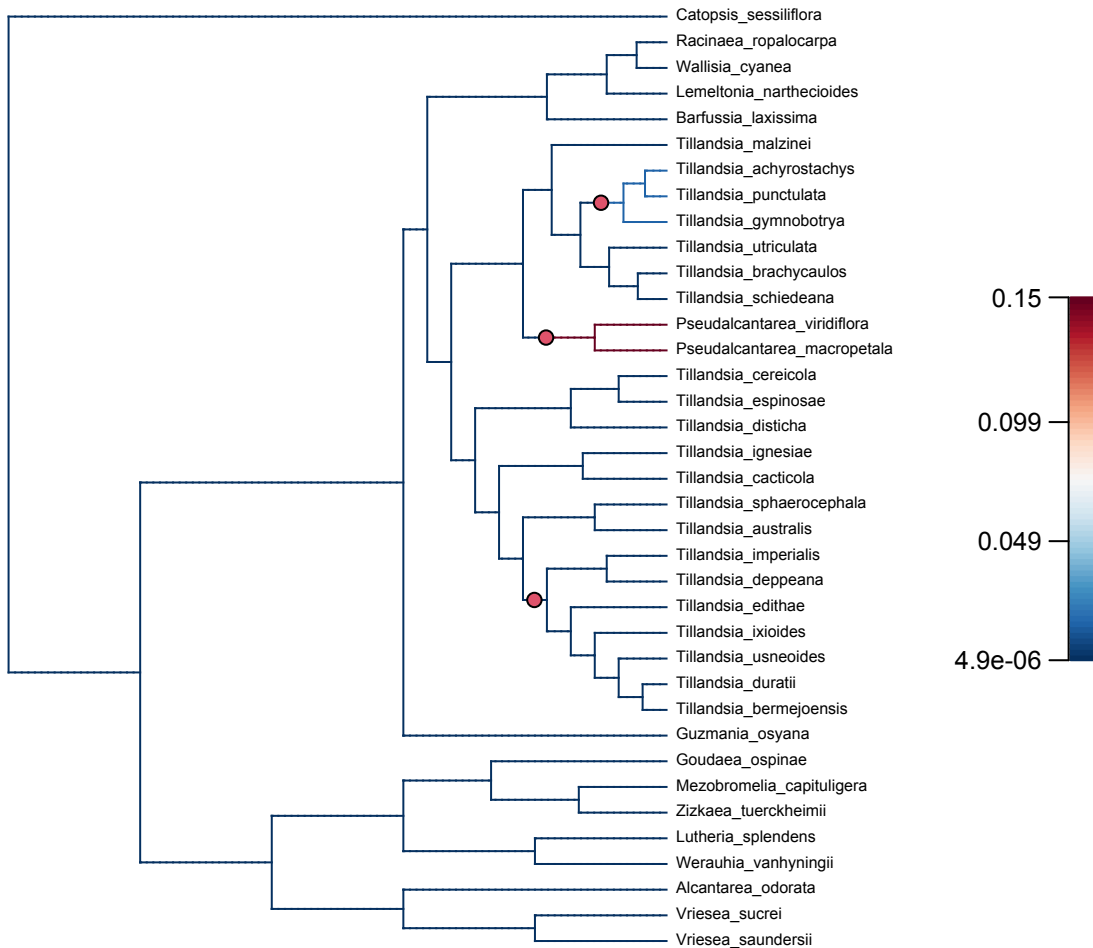

Supplement: Supplementary Material 7 — BAMM results for six plastome attributes where evolutionary rate shift sets were detected with PP ≥ 0.5. Branch colors reflect the rate of the trait evolution. [file Data_Sheet_3.ZIP › Supplementary_Material_S7/IR_size.pdf]

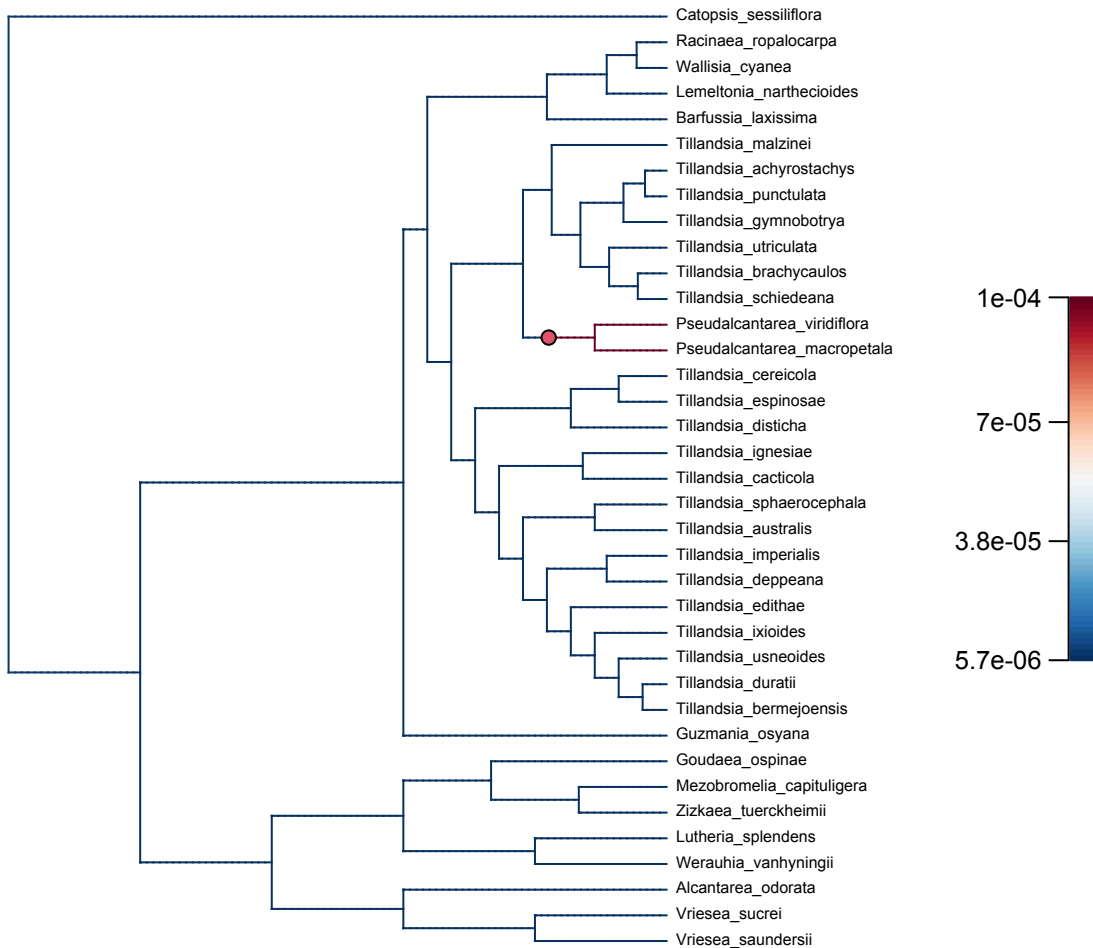

Supplement: Supplementary Material 7 — BAMM results for six plastome attributes where evolutionary rate shift sets were detected with PP ≥ 0.5. Branch colors reflect the rate of the trait evolution. [file Data_Sheet_3.ZIP › Supplementary_Material_S7/LSC_GC_content.pdf]

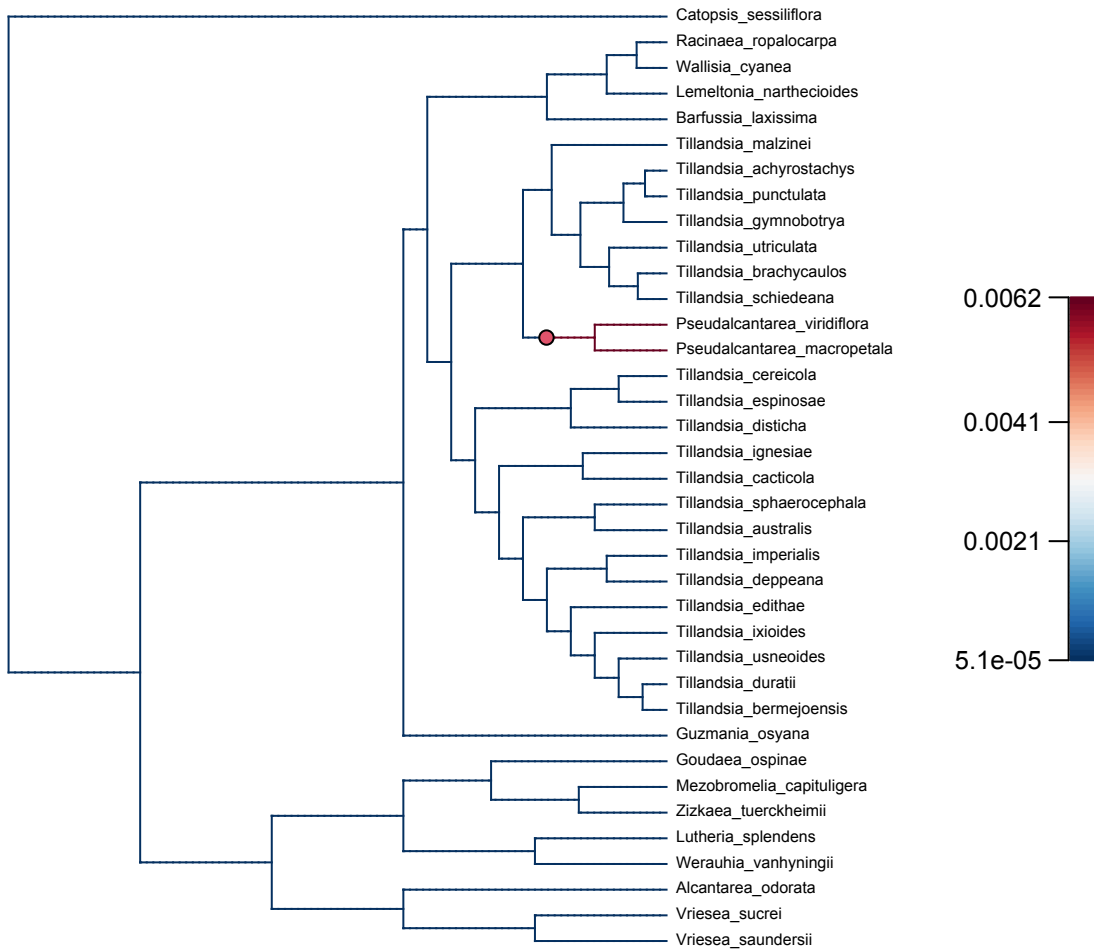

Supplement: Supplementary Material 7 — BAMM results for six plastome attributes where evolutionary rate shift sets were detected with PP ≥ 0.5. Branch colors reflect the rate of the trait evolution. [file Data_Sheet_3.ZIP › Supplementary_Material_S7/LSC_size.pdf]

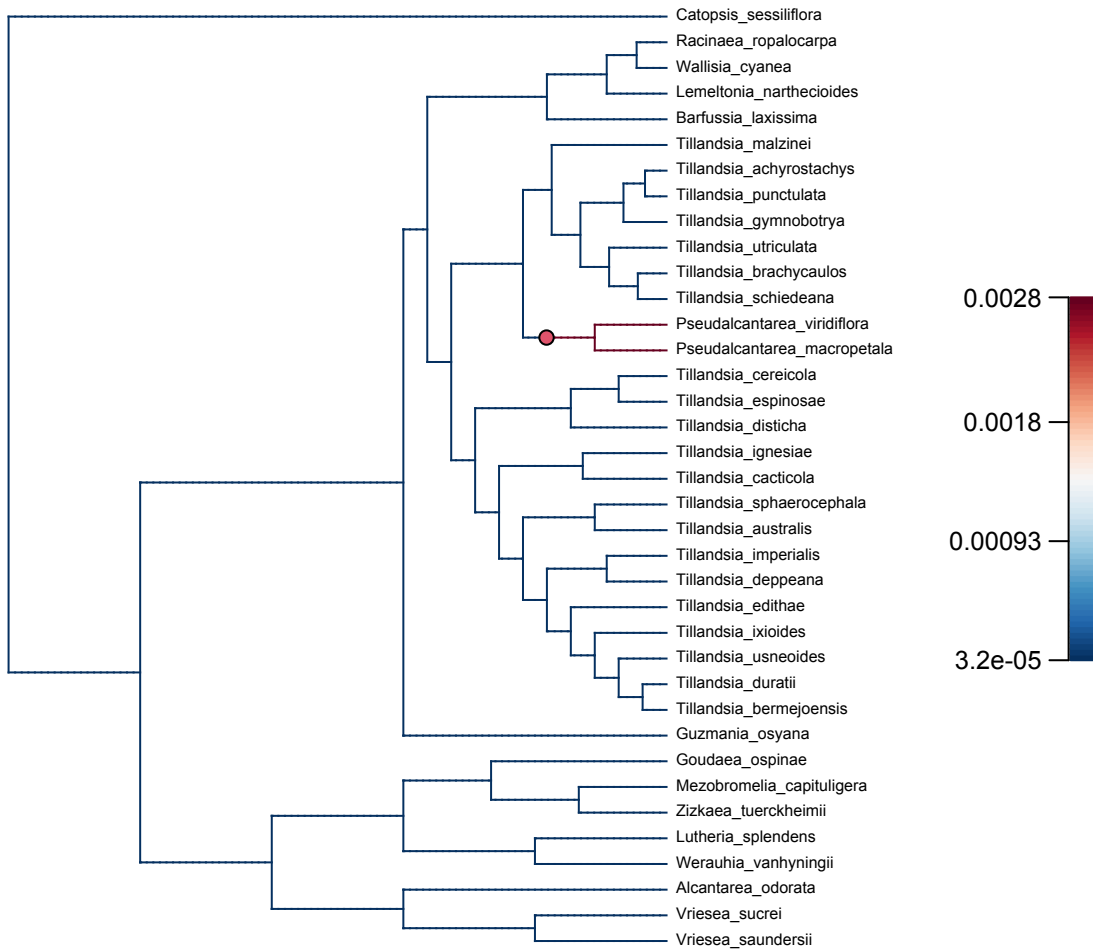

Supplement: Supplementary Material 7 — BAMM results for six plastome attributes where evolutionary rate shift sets were detected with PP ≥ 0.5. Branch colors reflect the rate of the trait evolution. [file Data_Sheet_3.ZIP › Supplementary_Material_S7/Plastome_size.pdf]

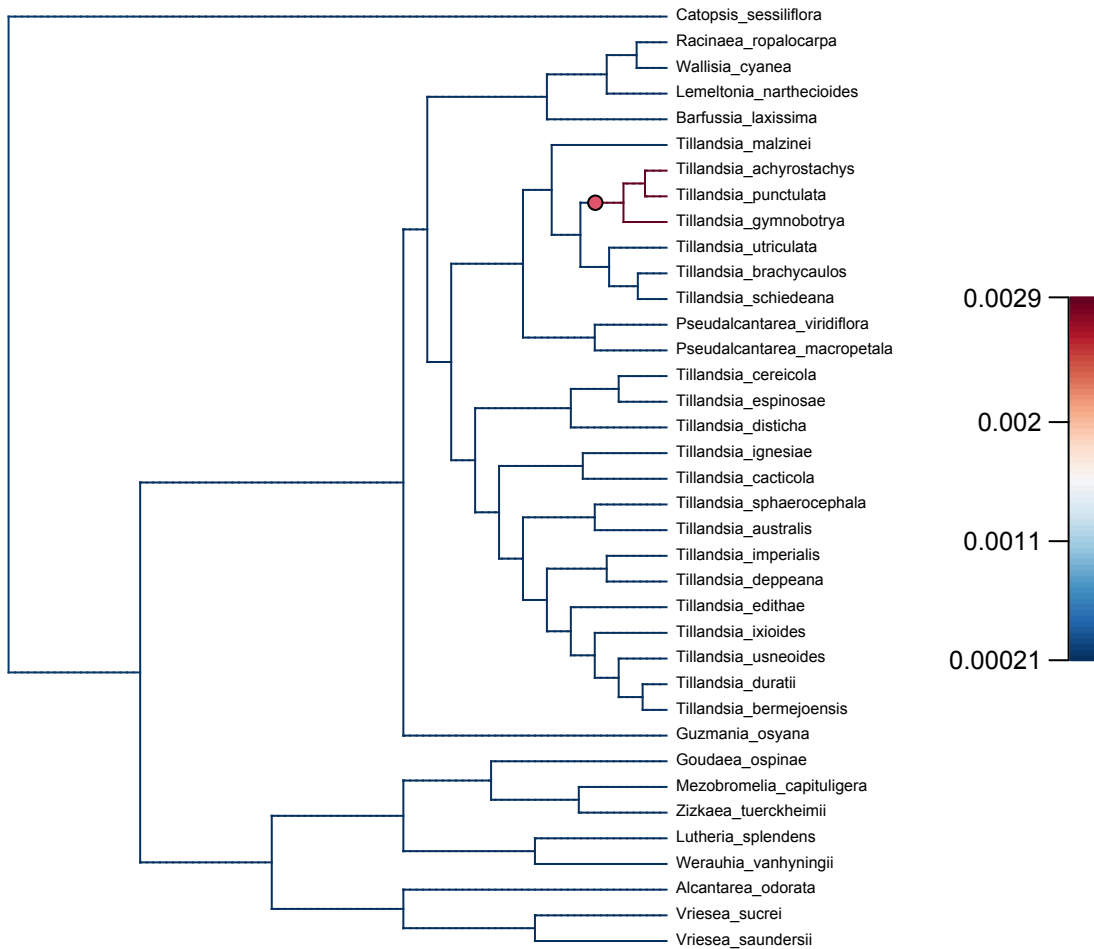

Supplement: Supplementary Material 7 — BAMM results for six plastome attributes where evolutionary rate shift sets were detected with PP ≥ 0.5. Branch colors reflect the rate of the trait evolution. [file Data_Sheet_3.ZIP › Supplementary_Material_S7/SSC_size.pdf]
